# Evaluation of Heparin-Binding Protein as a novel biomarker for the detection of periprosthetic joint infection

**DOI:** 10.3389/fcimb.2025.1651759

**Published:** 2025-12-11

**Authors:** Haotian Zhou, Qianshui Hu, Rui Zhang, Yaji Yang, Feilong Li, Jianye Yang, Runxing Kang, Leilei Qin, Cheng Chen, Wei Huang

**Affiliations:** 1 Department of Orthopaedic Surgery, The First Affiliated Hospital of Chongqing Medical University, Chongqing, China; 2 Chongqing Municipal Health Commission Key Laboratory of Musculoskeletal Regeneration and Translational Medicine, Chongqing, China; 3 Orthopaedic Research Laboratory of Chongqing Medical University, Chongqing, China; 4 Chongqing Medical University, Chongqing, China; 5 Department of Orthopedics, Chongqing University Fuling Hospital, Chongqing, China

**Keywords:** Heparin-Binding Protein (HBP), periprosthetic joint infection (PJI), serum biomarkers, neutrophil, diagnostic accuracy study

## Abstract

**Background:**

Heparin-Binding Protein (HBP), released during neutrophil activation and degranulation, functions in antimicrobial defense, vascular integrity regulation, and immune signal amplification. As a key effector of the innate immune system, HBP is rapidly released in response to infectious stimuli and plays a pivotal role in the pathogenesis of infectious diseases. This study aimed to evaluate the diagnostic value of HBP in periprosthetic joint infection (PJI) and compare its performance with commonly used inflammatory biomarkers.

**Methods:**

In this prospective study, 156 patients undergoing revision surgery for either aseptic loosening or PJI following joint arthroplasty were enrolled. Serum samples were collected within 24 hours preoperatively. Levels of HBP, C-reactive protein (CRP), erythrocyte sedimentation rate (ESR), interleukin-6 (IL-6), and procalcitonin (PCT) were measured. Receiver operating characteristic (ROC) curve analysis was performed to assess the diagnostic performance of each marker, and univariate logistic regression was used to evaluate their association with PJI.

**Results:**

Serum HBP levels were significantly higher in the PJI group compared to the aseptic group (P < 0.001). The area under the ROC curve (AUC) for HBP in diagnosing PJI was 0.968 (95% CI: 0.943–0.993), outperforming CRP (0.760, 95% CI: 0.680–0.840), ESR (0.825, 95% CI: 0.753–0.896), IL-6 (0.875, 95% CI: 0.816–0.935), and PCT (0.663, 95% CI: 0.567–0.759). HBP also yielded the highest Wald χ² value (32.414) among all tested variables, with the clearest discrimination between groups in the fitted model.

**Conclusion:**

This study demonstrates that HBP is a superior diagnostic biomarker for PJI compared to traditional inflammatory indicators, offering higher sensitivity and greater cost-effectiveness. Its diagnostic advantage lies in its ability to rapidly reflect early neutrophil activation and immune initiation at the onset of infection, enabling earlier detection than conventional markers such as CRP and ESR. Given its simplicity, low cost, and strong diagnostic utility, HBP is particularly valuable for early screening of indolent infections caused by low-virulence pathogens, where traditional markers may fail.

## 1 Introduction

Periprosthetic joint infection (PJI) is one of the most serious complications following joint arthroplasty, with an incidence of approximately 0.5%–2% (Patel, 2023; Qin et al., 2020). Due to the biological characteristics of the causative microorganisms, PJI is often characterized by a prolonged disease course and complex treatment (Masters et al., 2022; Zhou et al., 2024; Owaid and Al-Ouqaili, 2025). The direct hospitalization cost is more than twice that of aseptic revision, and the recurrence rate remains high, with some severe cases even requiring multiple surgeries or amputation (Premkumar et al., 2021; Kurtz et al., 2022). Therefore, accurate diagnosis is a key strategy to reduce the clinical burden and adverse outcomes associated with PJI. Multiple biomarkers in synovial fluid have been demonstrated to possess high diagnostic value. With the advancement of pathogen detection technologies, emerging molecular diagnostic tools such as polymerase chain reaction (PCR) and next-generation sequencing (NGS) have also shown excellent performance in the detection of PJI pathogens, significantly advancing the development of precision diagnostics (Li et al., 2024; Rajput et al., 2022; Olearo et al., 2025; Kullar et al., 2023; Al-Ouqaili et al., 2020). However, considering the differences in resource availability, testing costs, and technical expertise across medical institutions, the widespread implementation of such technologies in clinical practice still faces considerable challenges (Qin et al., 2023).

Against this backdrop, hematological markers remain the most commonly used diagnostic tools in clinical practice due to their accessibility, broad applicability, and cost-effectiveness (Akcaalan et al., 2022; Yilmaz et al., 2023). However, conventional serum inflammatory markers such as C-reactive protein (CRP) and erythrocyte sedimentation rate (ESR) primarily reflect systemic inflammatory status and are limited by suboptimal sensitivity and specificity (Saleh et al., 2018; Yang et al., 2024). Cytokines such as interleukin-6 (IL-6), while more dynamic, are hampered by high assay complexity and poor stability, limiting their utility in resource-limited or primary care settings (Schindler et al., 2023). Therefore, there is an urgent need for a serological biomarker that is sensitive, specific, economically viable, and adaptable across different tiers of the healthcare system. Such a marker would greatly enhance early detection of PJI and improve clinical outcomes.

Neutrophils play a central role in the pathogenesis of PJI, acting as the primary effector cells responsible for sensing pathogens, limiting their proliferation and spread, and initiating the inflammatory response (Liew and Kubes, 2019; Amulic et al., 2012). They constitute the first line of defense against microbial invasion (Zhu et al., 2017; Raz et al., 2017)Among the mediators released during neutrophil degranulation, Heparin-Binding Protein (HBP) is a key inflammatory mediator released during the degranulation of neutrophils (Ipek et al., 2018). It can directly disrupt bacterial membrane integrity and enhance host immune responses by promoting the release of cytokines such as IL-6 (Pereira et al., 1990; McAuley et al., 2013; Linder et al., 2009). *In vivo*, this process is reflected by a rapid increase in serum HBP levels, which typically rise significantly within one hour after infection onset and return to normal within two to three days once the infection is controlled or resolved (Linder et al., 2012). As a marker of early neutrophil activation, HBP offers dynamic responsiveness to infection. Owing to its high sensitivity, rapid kinetics, and temporal resolution, HBP has shown superior diagnostic performance compared to traditional markers such as C-reactive protein (CRP) in a range of infectious diseases, including sepsis and pneumonia (Taha et al., 2024; Kyriazopoulou et al., 2024; Bateman et al., 2016). A growing body of evidence supports its value as a sensitive biomarker for the early detection and risk stratification of bacterial infections, providing clinicians with a valuable tool for timely clinical decision-making (Kong et al., 2022; Widén et al., 2023).

However, there has been no systematic evaluation of the application of HBP in the diagnosis of PJI. To date, only one preliminary study has suggested a potential diagnostic value of HBP in post-traumatic orthopedic infections. However, among the 222 patients enrolled in the experimental group, only 39 were confirmed cases of PJI, and the diagnostic performance of HBP in this subgroup was neither separately analyzed nor validated (Hemmann et al., 2024). Therefore, as a non-invasive, easily accessible, and potentially valuable biomarker, serum HBP still holds significant promise for further investigation in the context of PJI. In this prospective diagnostic accuracy study, serum samples were collected at a single preoperative time point to compare inflammatory marker levels between patients with PJI and those with aseptic loosening. Logistic regression analysis was used to quantify the associations of HBP with common serum inflammatory biomarkers, including CRP, ESR, PCT, and IL-6, and to further evaluate the diagnostic performance of HBP. The study was conducted at the First Affiliated Hospital of Chongqing Medical University (Chongqing, China), where data collection and analysis were performed without imposing additional financial burden or invasive procedures on the patients.

## 2 Methods

### 2.1 Patient cohort and characteristics

Patients were categorized into either the aseptic loosening group or the infection group based on the diagnostic criteria established by the Musculoskeletal Infection Society (MSIS) in 2014 (Parvizi et al., 2014). The diagnosis of periprosthetic joint infection (PJI) strictly followed these criteria. This prospective cohort study was conducted between June 2021 and December 2024 and included a total of 156 patients undergoing revision total hip or knee arthroplasty due to either PJI or aseptic failure. All enrolled patients were followed for a period of two years. Written informed consent was obtained from each participant prior to inclusion. The study protocol was approved by the institutional ethics committee (Approval No. 2021-258) and registered with the Chinese Clinical Trial Registry (Registration No. ChiCTR2100050785).

To ensure the accuracy of group classification and minimize potential confounding factors, strict exclusion criteria were established (Qin et al., 2020). Prior antibiotic treatment may reduce the diagnostic sensitivity for PJI, thereby increasing the risk of false-negative classification. In addition, patients with concurrent inflammatory joint diseases or those receiving immunosuppressive therapy may exhibit abnormal fluctuations in systemic inflammatory markers, including CRP, ESR, IL-6, and PCT, which could compromise the reliability of biomarker-based analysis. Therefore, patients were excluded if they had used antibiotics within the past two weeks, skin ulcers, hematoma, or other cutaneous infections within two weeks prior to enrollment; recent lower limb trauma, surgery, or joint dislocation; known inflammatory joint diseases such as rheumatoid arthritis or gout; active infections in other organ systems or sexually transmitted diseases; vascular disorders of the lower extremities (e.g., DVT, thromboembolism, or vasculitis); were undergoing immunosuppressive therapy; had morbid obesity (BMI > 35 kg/m²); or were diagnosed with active malignancy. Of the 156 initially enrolled patients, 135 met the eligibility criteria and were included in the final analysis—65 in the PJI group and 70 in the aseptic failure group (AF group). The remaining 21 patients were excluded for the following reasons: rheumatoid arthritis (n = 5), gout (n = 3), pneumonia (n = 2), deep vein thrombosis (n = 1), prostatitis (n = 1), lower limb skin ulcer (n = 2), tinea pedis (n = 1), malignancies (n = 2; including one case of lung cancer and one of prostate cancer), and morbid obesity with BMI > 35 kg/m² (n = 4).

### 2.2 Baseline information and sample collection

Baseline demographic and clinical data were recorded for all participants, including age, sex, body mass index (BMI), and the affected joint. Within 24 hours of hospital admission, peripheral venous blood samples were collected to measure serum levels of erythrocyte ESR, CRP, PCT, IL-6, and HBP. For serum collection, venous blood was drawn into clean, additive-free tubes and allowed to clot at room temperature for 30 minutes. The samples were then centrifuged at 800g for 20 minutes to separate the serum, which was immediately subjected to biomarker testing.

In this study, serum HBP levels were measured using a commercial ELISA kit provided by Shanghai XuanKe Biotechnology Co., Ltd. All procedures were performed strictly in accordance with the manufacturer’s instructions. After sample preparation, both reagents and serum samples were equilibrated to room temperature. To ensure that all measurements fell within the linear detection range of the kit (15–520 pg/mL), gradient pre-dilution (1:10, 1:20, 1:50, 1:100) was performed for each sample prior to formal testing. The preliminary dilutions were compared against the standard curve to determine which dilution yielded an optical density (OD) value within the linear range (correlation coefficient r ≥ 0.9900). This optimal dilution ratio was then used for the final measurement of each sample.After mixing the diluted samples with the buffer solution, 100 μL was added to the test cassette and incubated at room temperature for 15 minutes. OD values were then measured using a Multiskan FC microplate reader (Thermo Fisher Scientific, USA), and corresponding HBP concentrations were recorded. To ensure consistency and accuracy, all samples were tested using the same batch of reagents, the same instrument, and the same operator.

The assay performance characteristics of the kit were as follows: spike recovery rates ranged from 85% to 115%, indicating good accuracy; the linear detection range was 15–520 pg/mL; the correlation coefficient (r) of the standard curve was ≥0.9900, demonstrating a strong linear relationship between concentration and signal; the intra-assay coefficient of variation (CV) was <10%, and the inter-assay CV was <15%, indicating high reproducibility.

During revision surgery, at least three periprosthetic tissue samples were obtained from each patient and subjected to standard microbiological culture for 24–48 hours, as well as prolonged culture for up to 14 days.

### 2.3 Statistical analysis

All statistical analyses were performed using GraphPad Prism version 10.2.3 (GraphPad Software, San Diego, California, USA). Data were first tested for normality. Continuous variables with a normal distribution were expressed as mean ± standard deviation (SD), and comparisons between groups were made using the independent samples *t*-test. Non-normally distributed variables were reported as median with interquartile range [M (Q1, Q3)], and comparisons were conducted using the Mann–Whitney *U* test. Receiver operating characteristic (ROC) curve analysis was used to evaluate the diagnostic performance of CRP, ESR, PCT, IL-6, and HBP for identifying PJI. The area under the curve (AUC), sensitivity, and specificity were calculated for each marker. The optimal cut-off value for lymph node count on CT to differentiate PJI from aseptic loosening was determined using the Youden index (J = sensitivity + specificity − 1). DeLong’s test was used to compare the AUCs of different biomarkers. A *P*-value < 0.05 was considered statistically significant, with significance levels defined as follows: *P* < 0.05 (*), *P* < 0.01 (**), and *P* < 0.001 (***).

Prior to the formal commencement of this study, we estimated the required sample size using G*Power 3.1 software. Based on the effect sizes commonly observed in diagnostic studies involving biomarkers for infectious diseases (e.g., CRP, PCT, calprotectin), we preset the effect size (Cohen’s d) at 0.8, representing a large effect. The significance level (α) was set at 0.05, the statistical power (1–β) at 0.95, and an equal allocation ratio (1:1) between groups was assumed (Sejersen et al., 2025; Christensen et al., 2022). The calculation indicated that a minimum of 42 participants per group (total n = 84) would be required to detect the expected effect with 95% power at a 5% significance level, ensuring the statistical reliability and scientific validity of the study results.

## 3 Results

### 3.1 Baseline characteristics

**Table 1** summarizes the demographic and clinical characteristics of the two groups. A total of 135 patients were included in the study, with 65 patients diagnosed with PJI according to the MSIS criteria and 70 patients undergoing revision surgery due to aseptic failure (AF). There were no significant differences between the two groups in terms of age, height, weight, BMI, sex, or affected joint (P > 0.05).

**Table 1:** Demographic and clinical characteristics.

Characteristic	PJI group (N = 65)	Aseptic failure (N = 70)	*P* value
Age(years)	69.08 ± 11.00	66.14 ± 13.06	0.231
Weight(kg)	61.72 ± 9.41	60.25 ± 11.12	0.554
Height(cm)	160.33 ± 8.66	161.27 ± 8.67	0.583
BMI(kg/m2)	24.12 ± 3.99	23.11 ± 3.67	0.177
Gender			0.384
Male	24(36.92%)	31(44.29%)	
Female	41(63.08%)	39(55.71%)	
Joint			0.311
Knee	12(23.08%)	18(26.92%)	
Hip	53(76.92%)	52(73.08%)	

Continuous variables with a normal distribution are presented as mean ± standard deviation (SD), and categorical variables are presented as counts (percentages). Statistical significance was defined as P ≤ 0.05.

PJI, periprosthetic joint infection; BMI, body mass index.

### 3.2 Laboratory test results

As shown in **Table 2**, serum levels of CRP, ESR, PCT, IL-6, and HBP were compared between the PJI group and the AF group. Significant differences were observed in all five biomarkers (P < 0.05).The median ESR in the PJI group was 53.00 mm/h (IQR: 41.00–76.00), significantly higher than that in the AF group (24.00 mm/h, IQR: 15.00–44.50).Median CRP levels were 16.20 mg/L (IQR: 8.58–30.20) in the PJI group, compared to 6.95 mg/L (IQR: 3.22–12.94) in the AF group. Median PCT levels also differed significantly, with 0.47 ng/mL (IQR: 0.07–0.81) in the PJI group and 0.05 ng/mL (IQR: 0.03–0.20) in the AF group.IL-6 levels were markedly elevated in the PJI group, with a median of 1102.50 pg/mL (IQR: 521.00–2805.45), in contrast to 41.81 pg/mL (IQR: 20.94–93.62) in the AF group. Similarly, HBP levels were significantly higher in the PJI group, with a median of 26.13 ng/mL (IQR: 23.00–31.70), compared to 8.11 ng/mL (IQR: 5.06–13.02) in the AF group.

**Table 2:** Inflammatory markers in patients with PJI and aseptic failure.

Biomarkers	Aseptic failure (N = 70)	PJI (N = 65)	*P* value
CRP	6.95 (3.22, 12.94)	16.20 (8.58, 30.20)	<0.001
PCT	0.05 (0.03, 0.20)	0.47 (0.07, 0.81)	0.001
ESR	24.00 (15.00, 44.50)	53.00 (41.00, 76.00)	<0.001
IL-6	41.81 (20.94, 93.62)	1102.50 (521.00,2805.45)	<0.001
HBP	8.11 (5.06, 13.02)	26.13 (23.00, 31.70)	<0.001

Non-normally distributed continuous variables are presented as median [IQR (Q1, Q3)], and categorical variables are presented as counts (percentages). Statistical significance was defined as P ≤ 0.05.

PJI, periprosthetic joint infection; CRP, C-reactive protein; PCT, procalcitonin; ESR, erythrocyte sedimentation rate; IL-6, interleukin-6; HBP, Heparin-Binding Protein.

### 3.3 Logistic regression analysis

**Figure 1:**
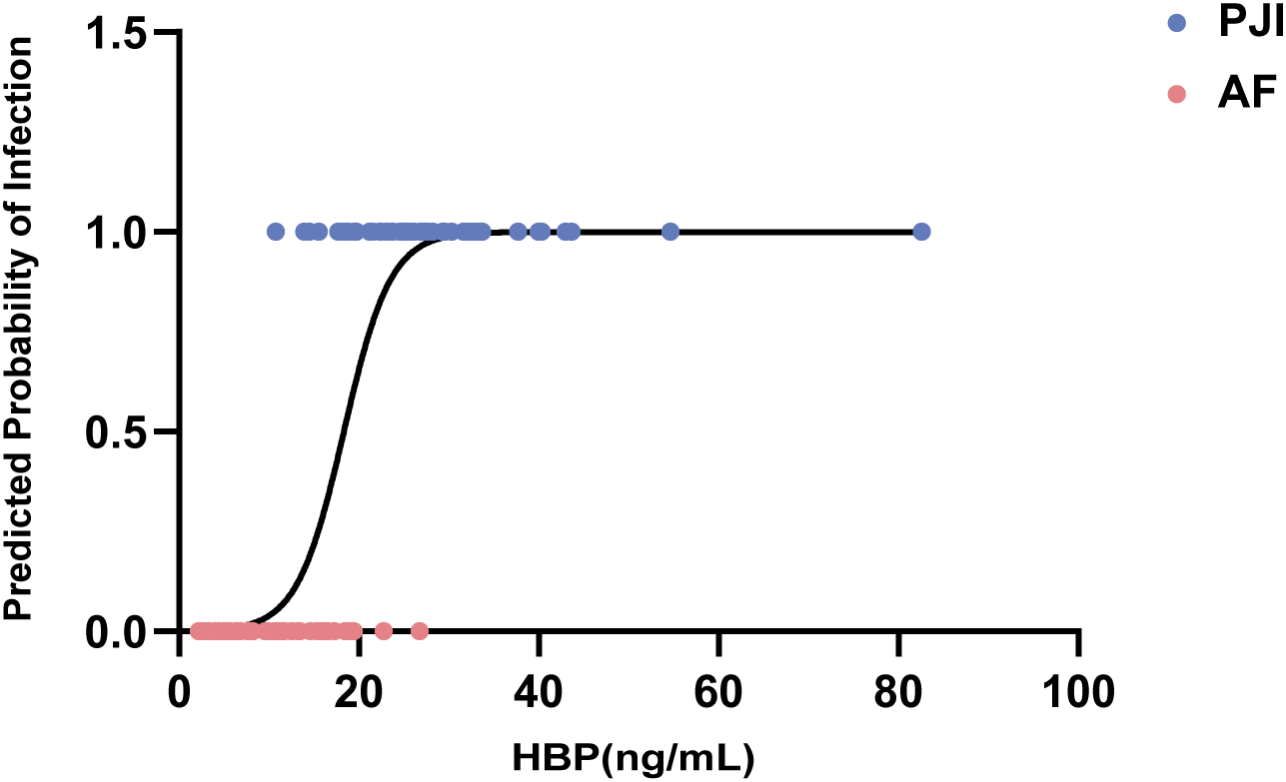
Probability of infection based on HBP levels (logistic regression model). HBP, Heparin-Binding Protein; PJI, periprosthetic joint infection; AF, aseptic failure.

Univariate logistic regression analysis was performed for five serum biomarkers—CRP, ESR, PCT, IL-6, and HBP—to assess their associations with PJI status (coded as 0 for aseptic failure and 1 for PJI) (**Table 3**). Among all variables, HBP demonstrated the most distinct classification boundary in the fitted regression curve. As HBP levels increased, the probability of being classified as PJI rose sharply. Notably, HBP yielded the highest Wald χ² value across all predictors (**Figure 1**), indicating the strongest statistical significance. Taken together, these findings suggest that among the five serum biomarkers evaluated in this study, HBP exhibits the greatest discriminatory power in differentiating PJI from aseptic failure.

**Table 3:** Logistic regression analysis of inflammatory markers.

Biomarkers	β	SE	Wald χ²	*P* value	OR	95%*CI*
CRP	0.0399	0.0124	10.368	0.001	1.044	1.019 to 1.070
ESR	0.0598	0.0112	28.508	<0.001	1.062	1.040 to 1.087
PCT	0.4373	0.233	3.5225	0.0605	1.549	1.025 to 2.591
IL-6	0.0017	0.0004	18.455	<0.001	1.002	1.001 to 1.003
HBP	0.3843	0.0675	32.414	<0.001	1.469	1.311 to 1.717

SE, standard error; OR, odds ratio; β, regression coefficient; 95% CI, 95% confidence interval.

PJI, periprosthetic joint infection; CRP, C-reactive protein; PCT, procalcitonin; ESR, erythrocyte sedimentation rate; IL-6, interleukin-6; HBP, Heparin-Binding Protein.

### 3.4 Receiver operating characteristic curve analysis

**Figure 2:**
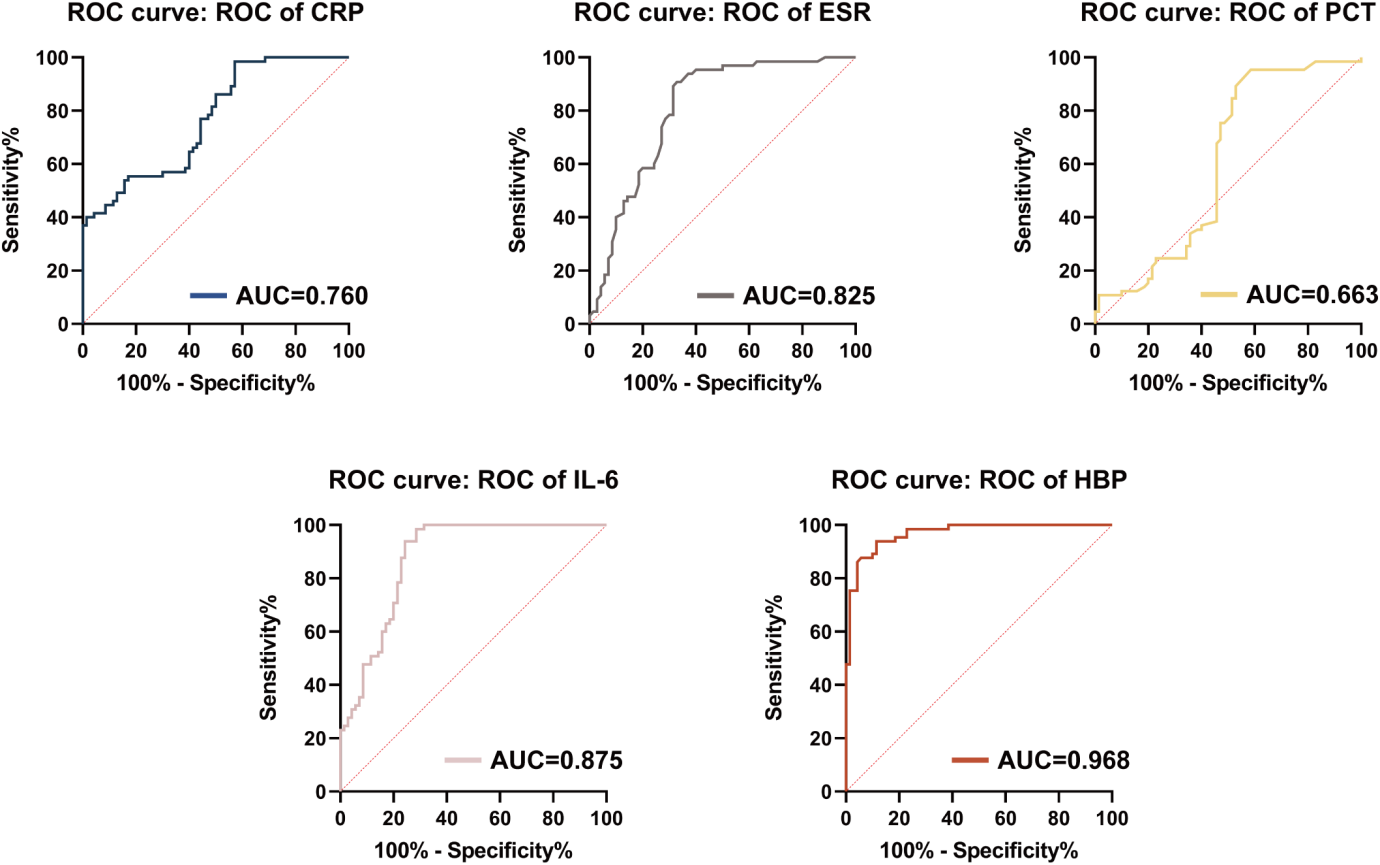
Receiver operating characteristic (ROC) curves and area under the curve (AUC) for five tests in the diagnosis of PJI. CRP, C-reactive protein; PCT, procalcitonin; ESR, erythrocyte sedimentation rate; IL-6, interleukin-6; HBP, Heparin-Binding Protein.

To further evaluate the discriminatory power of HBP in differentiating PJI from AF, we constructed receiver operating characteristic (ROC) curves using PJI patients as the positive group and AF patients as the negative group (**Figure 2**). The diagnostic performance of each inflammatory marker was assessed (**Table 4**). HBP demonstrated the highest diagnostic efficacy among all tested biomarkers, with an area under the ROC curve (AUC) of 0.968 (95% CI: 0.943–0.993). The optimal diagnostic cutoff was determined to be 17.45 ng/mL, yielding a sensitivity of 93.85%, specificity of 81.58%, and an overall diagnostic accuracy of 91.11%. IL-6 and ESR exhibited moderate diagnostic performance, with AUCs of 0.875 (95% CI: 0.816–0.935) and 0.825 (95% CI: 0.753–0.896), respectively. The sensitivity and specificity of IL-6 were 87.69% and 84.29%, while those of ESR were 83.08% and 77.14%, respectively. In contrast, CRP and PCT showed relatively lower diagnostic accuracy. The AUC for CRP was 0.760 (95% CI: 0.680–0.840), with an overall accuracy of 67.41%. For PCT, the AUC was 0.663 (95% CI: 0.567–0.759), with an accuracy of 69.63%. Statistical comparisons using DeLong’s test revealed that the AUC of HBP was significantly higher than those of CRP, ESR, PCT, and IL-6 (P < 0.05), indicating that serum HBP possesses superior overall diagnostic performance in identifying PJI.

**Table 4:** Diagnostic performance parameters of five biomarkers for PJI.

Biomarkers	AUC (95% CI)	Sensitivity, % (95% CI)	Specificity, % (95% CI)	Cutoff Value	PPV, %	NPV, %	Accuracy, %
CRP	0.760 (0.680 to 0.840)	81.52 (75.73 to 92.54)	64.29 (35.90 to 58.68)	12.85	68.42	66.67	67.41
ESR	0.825 (0.753 to 0.896)	83.08 (72.18 to 90.28)	77.14 (66.05 to 85.41)	31.50	71.95	88.68	78.52
PCT	0.663 (0.567 to 0.759)	76.92 (65.36 to 85.49)	62.86 (51.15 to 73.23)	0.065	65.79	74.58	69.63
IL-6	0.875 (0.816 to 0.935)	87.69 (77.55 to 93.63)	84.29 (74.01 to 90.99)	237.7	76.19	98.04	84.44
HBP	0.968 (0.943 to 0.993)	93.85 (85.22 to 97.58)	81.58 (79.04 to 94.09)	17.45	88.41	93.94	91.11

PJI, periprosthetic joint infection; CRP, C-reactive protein; PCT, procalcitonin; ESR, erythrocyte sedimentation rate; IL-6, interleukin-6; HBP, Heparin-Binding Protein.

## 4 Discussion

PJI remains one of the most challenging complications in orthopedic surgery. In recent years, several authoritative organizations, including the European Bone and Joint Infection Society (EBJIS) and the Musculoskeletal Infection Society (MSIS), have proposed and continuously updated diagnostic criteria for PJI (McNally et al., 2021; Parvizi et al., 2018). Nevertheless, no single test has yet achieved 100% diagnostic accuracy. In this prospective study, we evaluated the diagnostic performance of HBP, a novel serum biomarker, for the detection of PJI. Our findings demonstrate that HBP is a sensitive and reliable indicator of PJI. Compared with traditional inflammatory markers such as CRP, ESR, IL-6, and PCT, HBP exhibited higher sensitivity and specificity. These results highlight its potential clinical utility for early identification of PJI and for guiding timely intervention strategies.

Elevated levels of HBP have been reported as important evidence in the diagnosis of infectious diseases such as sepsis and pneumonia. Jonas Sundén-Cullberg et al. found that approximately 87% of patients with septicemia exhibited elevated serum HBP levels, and this elevation was associated with increased mortality risk (Linder et al., 2012). Similarly, a study by Li et al. demonstrated the diagnostic potential of HBP in hospital-acquired pneumonia (HAP), where HBP levels were significantly higher in HAP patients than in controls, with an AUC of 0.711 (Wang et al., 2022). Consistent with these findings, our study also observed varying degrees of HBP elevation in patients with PJI. This phenomenon may reflect a robust activation of neutrophils during the pathophysiological process of PJI and further supports the potential role of HBP as a diagnostic biomarker in deep-seated infections such as PJI.

Neutrophils are key effector cells of the innate immune system that eliminate pathogens in the early stages of infection through degranulation, phagocytosis, and the formation of NETs (Amulic et al., 2012; Lehrer et al., 1988; Al-Ouqaili and Ismael) Given these mechanisms, biomarkers associated with neutrophil activation are often considered reliable indicators of infection onset. NETs formation represents a stress-induced antimicrobial mechanism employed by neutrophils in response to bacterial invasion (Papayannopoulos, 2018; Hidalgo et al., 2022). Medina et al. reported that NETs-associated plasma markers possess potential predictive value for PJI (Oto et al., 2024). However, it is noteworthy that NET formation depends on ROS signaling activation, and certain pathogens such as *Staphylococcus aureus* can suppress ROS generation by upregulating IL-10 and promoting mitophagy. This mechanism implies that the diagnostic applicability of NETs-related biomarkers may be limited in *S. aureus* infections (Zhan et al., 2023; Wise et al., 2025; Yang et al., 2025).

In contrast, biomarkers released during neutrophil degranulation may offer greater advantages for early infection detection due to their earlier release kinetics and more stable regulatory mechanisms (Lodge et al., 2020). HBP is a neutrophil-derived granule protein with combined bactericidal activity, chemotactic properties, and immunomodulatory functions. Its release is closely associated with the onset and progression of infection (Kolaczkowska and Kubes, 2013). Our study demonstrated that HBP outperformed not only conventional inflammatory markers such as CRP and ESR, but also IL-6 and another neutrophil-associated marker, PCT. Unlike PCT, which is synthesized in the liver in response to cytokine signaling, HBP is rapidly released from activated neutrophils, allowing for a swifter response to infection (Pereira et al., 1990; McAuley et al., 2013; Qu et al., 2015; Schuetz et al., 2013). In contrast to IL-6, HBP acts at an earlier stage of immune activation, serving as a more direct indicator of neutrophil activity, thereby offering enhanced specificity and stability (Linder et al., 2009). Supporting evidence was reported by Zhou et al., who found that in the diagnosis of septicemia, HBP had a significantly higher AUC (0.88) than both IL-6 (0.59) and PCT (0.78) (Mao et al., 2025). In our study, univariate logistic regression analysis further confirmed the strong association between HBP and PJI. Among all evaluated markers, HBP demonstrated the most distinct classification boundary and yielded the highest Wald χ² value, reinforcing its superior diagnostic value in distinguishing PJI from aseptic failure.

This study has several limitations. First, the sample size was relatively limited, and all patients were recruited from a single institution, which may introduce selection bias. Second, patients with coexisting inflammatory conditions or other infections were excluded, potentially limiting the generalizability of HBP as a diagnostic biomarker in more complex clinical settings. Third, HBP levels were measured at a single time point, which may not fully capture its dynamic changes throughout the course of infection.

This study conducted a systematic evaluation of the clinical utility of HBP in the diagnosis of PJI. The findings indicate that HBP outperforms currently used inflammatory biomarkers in diagnostic accuracy. Owing to its excellent sensitivity, accessibility, and cost-effectiveness, HBP offers a promising tool for the early identification of PJI. However, this study has certain limitations, including a relatively homogeneous study population. Future research should aim to validate the diagnostic value and dynamic changes of HBP across various pathogens, stages of infection, and perioperative time points using multicenter, large-sample, and longitudinal data. Moreover, the potential for local HBP detection and combination with other biomarkers should be further explored to enhance the precision of PJI diagnosis.

## Data Availability

The raw data supporting the conclusions of this article will be made available by the authors, without undue reservation.
